# Non-surgical Pneumoperitoneum in the Setting of Gram-negative Sepsis

**DOI:** 10.7759/cureus.2493

**Published:** 2018-04-17

**Authors:** Jacob A Sambursky, Sundeep Kumar, Molly Orban, Esteban Janolo, Vladimir Neychev

**Affiliations:** 1 University of Central Florida College of Medicine, Orlando, USA; 2 Internal Medicine, University of Central Florida College of Medicine, Orlando, USA; 3 Ophthalmology, Osceola Regional Medical Center, Kissimmee, USA; 4 Internal Medicine, Osceola Regional Medical Center, Kissimmee, USA; 5 Department of Clinical Sciences, University of Central Florida College of Medicine, Orlando, USA

**Keywords:** gram negative sepsis, non surgical pneumoperitoneum, spontaneous pneumoperitoneum, sub diaphragmatic air, surgical pneumoperitoneum

## Abstract

Pneumoperitoneum is described as the presence of free air in the peritoneal cavity. In the majority of cases, it is the manifestation of abdominal viscus perforation, requiring an emergent surgical exploration. In rare cases, however, no evidence of perforation of the gastrointestinal or genitourinary tracts can be found at exploration, and in such cases, the pneumoperitoneum is referred to as non-surgical pneumoperitoneum. We present a case of an 87-year-old man who developed a non-surgical pneumoperitoneum in the setting of gram-negative sepsis. The patient was admitted for treatment of obstructive uropathy and sepsis secondary to a gram-negative urinary tract infection. Despite the initial resuscitation and antibiotic therapy, his hospital course was complicated by worsening abdominal discomfort, and a chest radiograph revealed free air under the diaphragm. He was taken to the operating room for an emergent surgical exploration that revealed no visceral perforation or other possible surgical causes. He tolerated and recovered from surgery well, and had a complete resolution of pneumoperitoneum in the early post-surgery period, per radiographic imaging. This interesting case highlights a rare case of idiopathic nonsurgical pneumoperitoneum in the setting of gram-negative sepsis. Additionally, we discuss considering non-surgical etiologies for patients without clinical signs or surgical evidence of perforation.

## Introduction

Pneumoperitoneum, described as the presence of free air in the peritoneal cavity, generally mandates emergent surgical exploration in the majority of cases [1,2]. Only 10% of these cases are non-surgical pneumoperitoneum, also referred to as spontaneous or idiopathic pneumoperitoneum [1,3]. A diagnosis of non-surgical pneumoperitoneum is appropriate when surgical repair is not the indicated treatment for the known cause of pneumoperitoneum, or when an exploratory laparotomy is done and no perforation is found [1,2]. We present a case of non-surgical pneumoperitoneum in the setting of gram-negative septicemia.

## Case presentation

An 87-year-old Caucasian male with a past medical history of benign prostatic hyperplasia and irritable bowel syndrome presented to the hospital with urinary incontinence, diarrhea, abdominal pain, hypotension and altered mental status. A diagnosis of septic shock secondary to urinary tract infection was made on arrival based on symptomology and initial investigation. The patient had a history of lower abdominal pain for last two weeks. He visited his primary care physician and underwent a computed tomography (CT) scan of the abdomen and pelvis which showed hypertrophy of the prostate and bilateral hydronephrosis. The patient had progression of symptoms leading to hospitalization. On arrival to the hospital, the patient was hemodynamically stable but quickly decompensated. Vitals showed a blood pressure of 88/55 mmHg, heart rate of 143 beats per minute, respiratory rate of 20 breaths per minute, and temperature of 96.3°F. Initial pertinent laboratory findings included acute kidney injury with serum creatinine of 12 mg/dL (from a baseline of 1.2 mg/dL) and blood urea nitrogen (BUN) of 161 mg/dL. Labs demonstrated an anion gap metabolic acidosis secondary to lactic acidosis. Urinalysis showed evidence of infection, and blood and urine samples sent for cultures. Physical exam at arrival was significant for a minor distress, diaphoresis, enlarged and tender prostate, abdominal distension without tenderness to palpation, guarding, rebound tenderness, or abnormal dermatological findings. The white blood cell values from the day of surgery until discharge are detailed in Table 1.

**Table 1:** White blood cell (WBC) count.

Day of admission	WBC value (x 10^3^/uL)
Day 5	15.99
Day 6	11.92
Day 7	9.17
Day 8	7.26
Day 9	12
Day 10	12.66
Day 11	11.69

The patient received empiric intravenous antibiotics and fluid resuscitation in the emergency department along with placement of a urinary catheter to relieve urinary obstruction. Urinary catheter placement revealed gross hematuria, but hematuria resolved by the next day. Blood and urine cultures were positive for E. coli and initial antibiotics were deescalated to ceftriaxone, to which the organism was sensitive.

**Figure 1:**
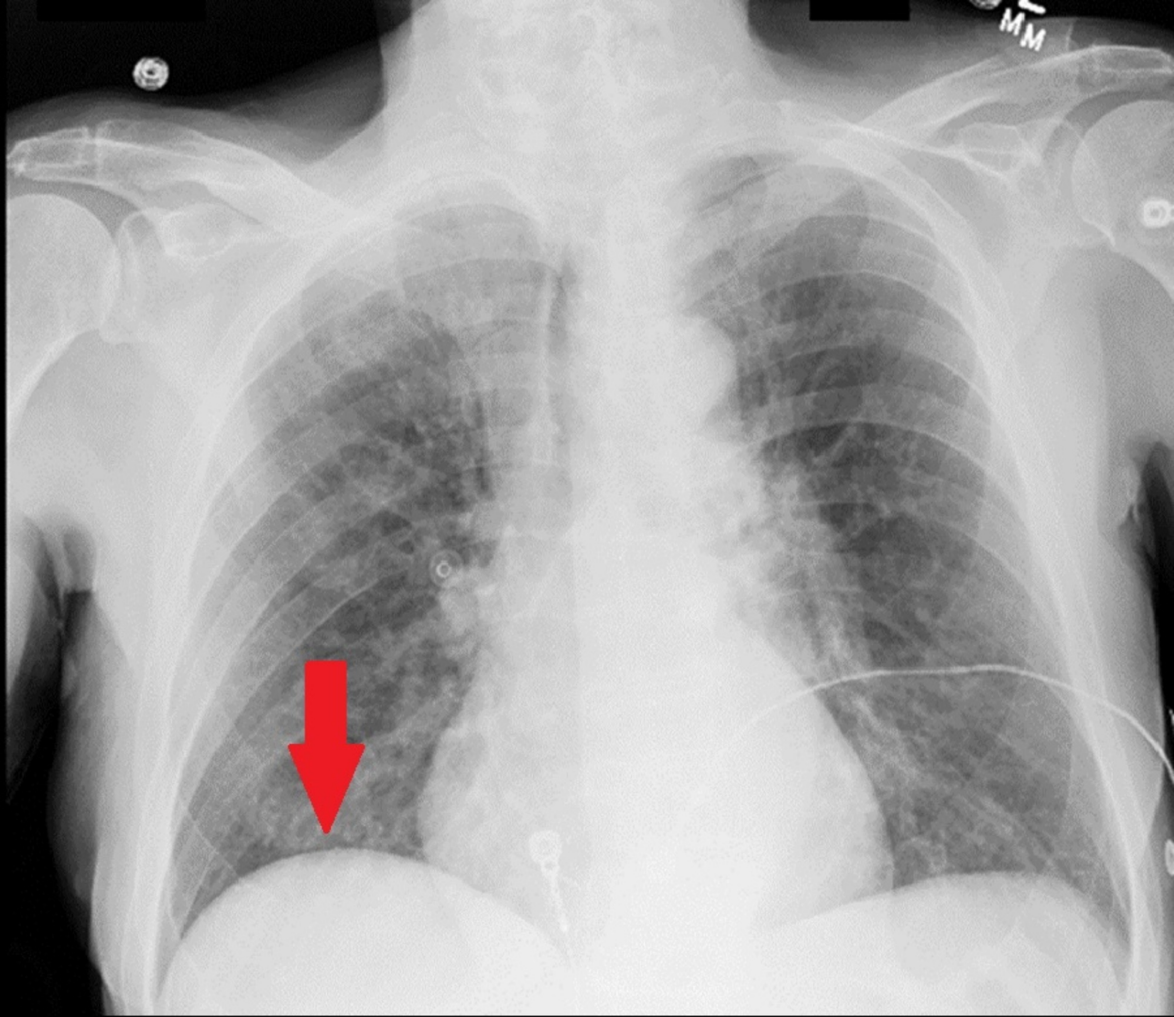
Chest radiograph, day 2 of admission. No radiographic evidence of acute cardiopulmonary pathology. Electrocardiogram wire visualized.

No acute cardiopulmonary changes were visualized on chest X-ray taken three days prior (Figure 1).

**Figure 2:**
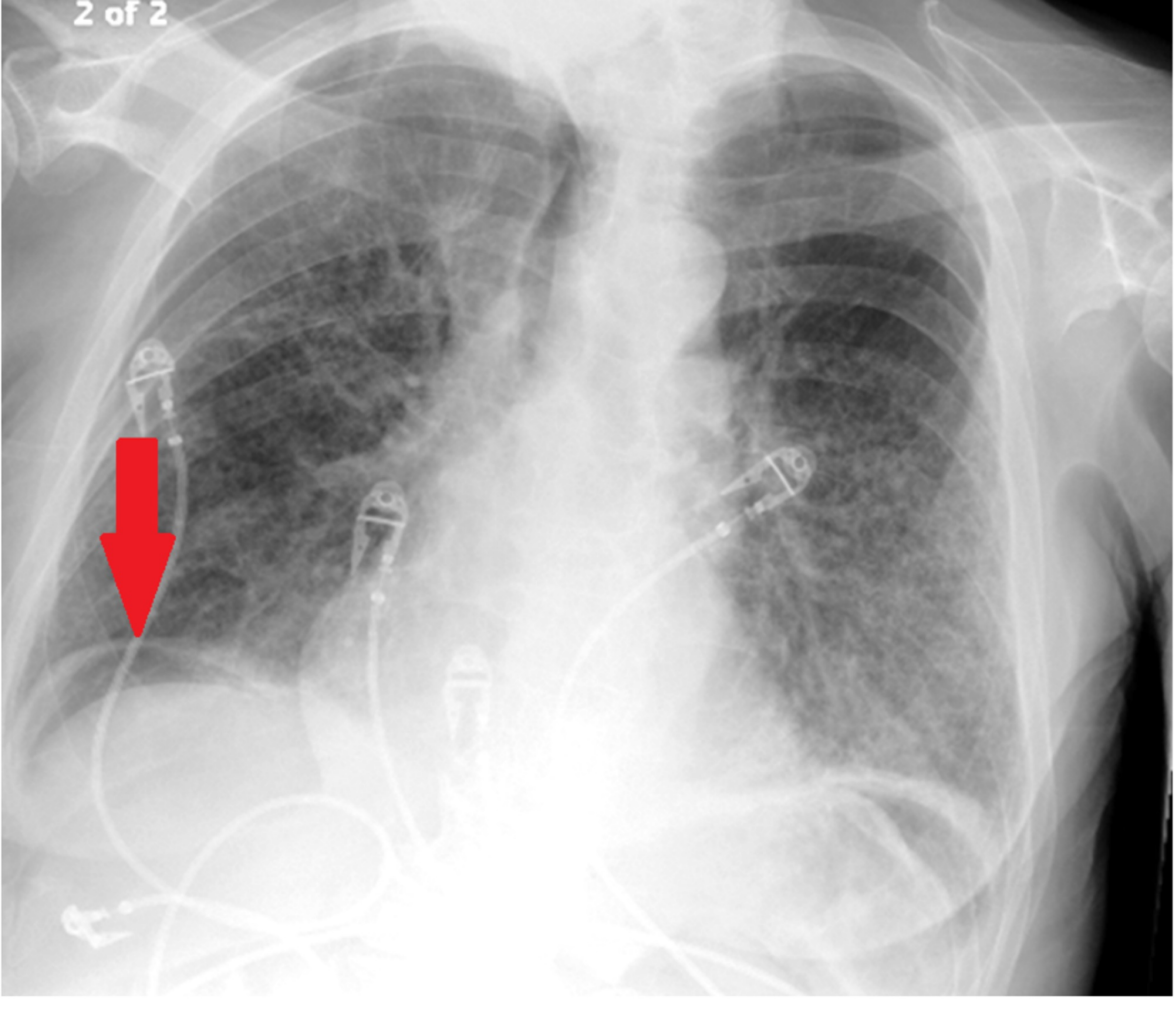
Chest radiograph, day 5 of admission. Radiologic upright film of the chest demonstrates right-sided, free intraperitoneal air (red arrow). Possible gastric bubble visualized cannot rule out left-sided, free intraperitoneal air. No evidence of other acute changes, such as enlargement of the mediastinum, cardiomegaly, pleural effusion, or pneumothorax. Electrocardiogram wires visualized.

On day five of his admission, the patient experienced increased abdominal pain, constipation, and subjective fevers. Vitals showed a temperature of 97.2°F, a blood pressure of 111/70 mmHg, a pulse of 87 beats per minute, respiratory rate of 20 respirations per minute, and oxygen saturation of 97%. Abdominal exam was significant for hypoactive bowel sounds, mild distension, guarding, tympany on percussion, and diffuse tenderness to palpation. An upright chest X-ray was ordered. Upright chest X-ray revealed free intraperitoneal air (Figure 2).

**Figure 3:**
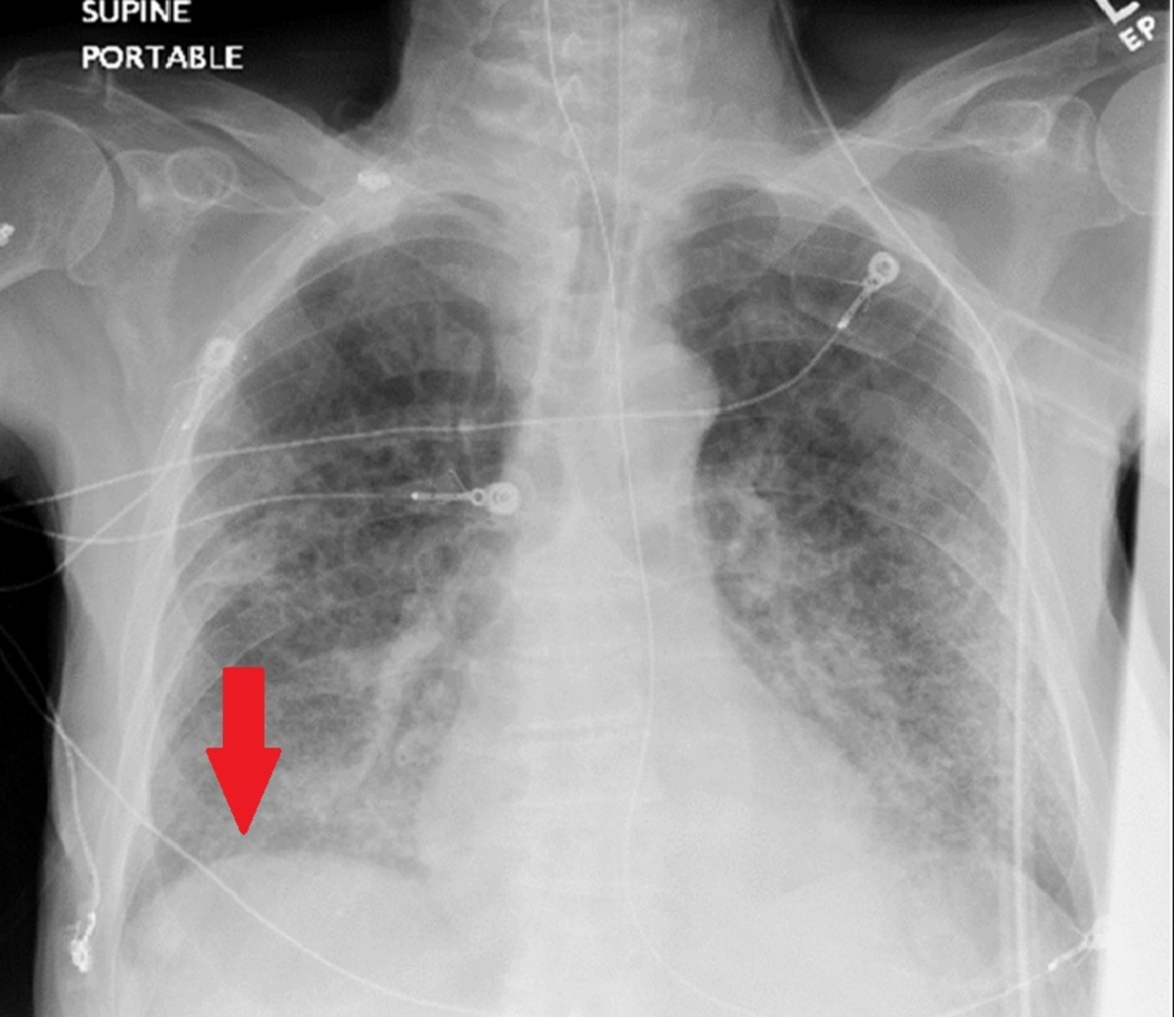
Chest radiograph, day 6 of admission. No evidence of acute cardiopulmonary pathology. No radiologic evidence of pneumoperitoneum. Electrocardiogram wires visualized.

An abdominal X-ray showed a nonobstructive bowel gas pattern without signs of dilated loops of bowel or air-fluid levels. The surgical team was emergently consulted for evaluation of pneumoperitoneum, and an urgent exploratory laparoscopy was recommended without further evaluation or imaging. The patient underwent emergent exploratory laparoscopy that was converted to laparotomy, as there was no evidence of a perforation visualized on laparoscopy. Meticulous examination of the abdominal cavity revealed no evidence of an intestinal or genitourinary tract perforation or any other surgical causes, peritonitis, free fluid, or abscess formation. Post-operative X-ray, completed on post-operation day one, demonstrated resolution of the pneumoperitoneum (Figure 3).

## Discussion

Pneumoperitoneum is the abnormal presence of air in the peritoneal cavity. Common symptoms associated with pneumoperitoneum are often consistent with acute peritonitis. Symptoms include anorexia, vomiting, bloating, and abdominal pain. Multiple imaging methods are available for the detection of pneumoperitoneum: chest radiography, abdominal radiography, and CT of the abdomen. The combination of abdominal pain and air under the diaphragm, even in the absence of other clinical signs, is usually enough for the surgical recommendation of explorative laparotomy to search for the causative lesion. The etiological spectrum is broad and includes both surgical and non-surgical causes from thoracic and abdominal processes or procedures, as well as urological, and gynecological conditions [2]. Cases of pneumoperitoneum with an unknown cause are termed idiopathic spontaneous pneumoperitoneum, and are extremely rare [4]. Common etiologies of surgical pneumoperitoneum include appendicitis (34%), diverticulitis (12%), and perforated peptic ulcer (10%) [4]. Non-surgical causes (~10%) include sepsis, pneumatosis cystoides intestinalis, pneumomediastinum (caused by esophageal or pulmonary pathology), oro-genital sexual intercourse, aerophagia, recent history of mechanical ventilation, cardiopulmonary resuscitation, and peritoneal dialysis [2]. If there is clinical suspicion or evidence of a potential surgical cause of pneumoperitoneum visualized on imaging, then exploratory laparotomy is the indicated treatment. However, in the scenario where there is no clinical suspicion for a surgical etiology and the patient is hemodynamically stable, then conservative management is a valid treatment option.

Kumar et al. reported visceral perforation causes 42% of cases of pneumoperitoneum, while 37% are caused by post-operative residual air [3]. Even in the modern-day era where CT scanners are widely available in emergency departments and provide invaluable etiologic data, conventional radiography using X-ray still holds diagnostic significance in the diagnosis of pneumoperitoneum. Studies showed that the upright chest radiograph holds a sensitivity of 71–98% in diagnosing pneumoperitoneum [5-7]. The sensitivity for free air detection was greatest with the use of lateral decubitus abdominal radiograph (98%) and upright chest radiograph (85.1%) [7]. However, the ability for free air to be detected on imaging is dependent on the cause and location of the perforation, if one exists [3]. CT carries a higher sensitivity than chest radiography and may help in localizing the source of the pneumoperitoneum. In a patient who is hemodynamically stable and presents with symptoms consistent with a perforation in the abdominal cavity, CT scan would be a better imaging modality. Studies have suggested extraluminal (free) gas in the sub-phrenic space as one of the most common radiological findings [2], first described by Popper in 1915. A study by Chiu et al. reported anterior superior oval sign as the most common finding in supine abdominal and chest radiography, and sub-phrenic radiolucency as the third most common finding on supine chest radiograph [7]. Other radiographic signs are detailed by Kumar et al. and Chiu et al. [3,7]. Surgical pneumoperitoneum typically presents with signs of peritonitis, such as abdominal rigidity, abdominal tenderness, fever, leukocytosis, and/or elevated inflammatory markers. The management approach includes surgical exploration, intravenous fluids, nutritional support, administration of broad-spectrum antibiotics, and hyperbaric oxygen (if necrotizing enterocolitis is present) [8].

Exploratory laparotomy is typically performed in patients with radiographic pneumoperitoneum, abdominal pain, and signs of peritonitis [2]. In cases of pneumoperitoneum where the etiology is believed to be non-surgical in nature, conservative treatment has been proposed to prevent unnecessary laparotomy [4]. Conservative treatment includes but not limited to parenteral nutrition, intravenous fluid resuscitation, intravenous antibiotics, and serial abdominal examinations and imaging to document resolution [8]. The difficulty lies in identifying the patients in whom conservative management is indicated. Though rare, non-surgical pneumoperitoneum must be considered as the etiology in patients with sepsis, recent mechanical ventilation or peritoneal dialysis, and other potential causes listed above in order to avoid unnecessary surgical exploration.

There are many proposed etiologies to explain the presence of pneumoperitoneum on radiographic imaging, as mentioned above. Iatrogenic causes were immediately dismissed for our patient as there was no recent history of positive airway pressure use, cardiopulmonary resuscitation, mechanical ventilation, peritoneal dialysis, or invasive procedures (endoscopy, colonoscopy, laparoscopy, laparotomy). Urogenital injury must be considered due to the traumatic Foley catheter insertion with gross hematuria in the emergency department. However, since there was no obvious injury and there was no free fluid in the abdomen at exploration, this etiology is less likely.

One potential, less likely, non-surgical etiology considered was Chilaiditi syndrome, where the transverse colon is interposed between the liver and diaphragm. This could potentially be misinterpreted as radiological evidence of a pneumoperitoneum. However, this was ruled out by the lack of haustra visualized in the right upper quadrant and the findings on exploratory laparotomy.

The final potential etiology is that our patient developed non-surgical pneumoperitoneum secondary to sepsis caused by documented gram-negative, gas-forming bacteria (Escherichia coli). There is no objective evidence of any other etiology that may have resulted in a non-surgical pneumoperitoneum. One case report described gram-negative bacteremia as the causative agent of pneumoperitoneum [9]. It is probable that this etiology involves a micro-perforation of the intestinal tract caused by inflammatory mediators in the setting of an infection. Similar micro-perforations have been reported to cause pneumoperitoneum after colonoscopy and intestinal procedures, like endoscopic retrograde cholangiopancreatography.

Chandler et al. report that 28% of patients with non-surgical pneumoperitoneum were subjected to surgical exploration. They report three recurring themes amongst these patients: a decision to perform exploratory surgery completely based on radiological evidence, radiolucency consistent with pneumoperitoneum not located at the apex of the diaphragm, and the clinical presence of marginal peritoneal symptoms [10].

Recognition of the potential for non-surgical pneumoperitoneum is important in preventing unnecessary surgical procedures that expose patients to infection, complications, and extended recovery periods. Consideration should be made for close evaluation of radiologic findings in cases where a clear surgical cause of pneumoperitoneum does not exist, and evaluation of other potential causes undertaken. For the case of the surgeon who elects to take a patient for exploratory laparotomy and finds no evidence supporting a surgical etiology, it is acceptable to terminate surgical exploration after adequate inspection of the entire length of the small and large bowel. The recognition of non-surgical pneumoperitoneum at the bedside and further insight into its etiopathogenesis will likely lead to improved morbidity and mortality.

## Conclusions

Our patient's clinical presentation and negative exploratory laparotomy imply a radiologically confirmed idiopathic pneumoperitoneum. Although the association between non-surgical pneumoperitoneum and gram-negative sepsis in this case is not impossible, the underlying pathophysiological mechanism is not yet understood. Further research is necessary to investigate the potential pathophysiological mechanisms. Overall, this case highlights the importance of careful consideration of clinical and radiographic findings in the diagnostic and therapeutic approach to pneumoperitoneum. Considering the potential causes of non-surgical pneumoperitoneum is important for the surgeon’s decision-making process for surgical versus non-surgical management. Recognition of non-surgical pneumoperitoneum may prevent unnecessary surgical procedures, like in the case of our patient, that contribute to morbidity and result in extended recovery periods. Further characterization of cases with non-surgical pneumoperitoneum is necessary to improve and solidify diagnostic criteria and allowing for effective, safe management and therapy.
